# BDNF-TrkB signaling-mediated upregulation of Narp is involved in the antidepressant-like effects of (*2R,6R*)-hydroxynorketamine in a chronic restraint stress mouse model

**DOI:** 10.1186/s12888-022-03838-x

**Published:** 2022-03-15

**Authors:** Lingsha Ju, Jiaojiao Yang, Tingting Zhu, Panmiao Liu, Jianjun Yang

**Affiliations:** 1 Department of Anesthesiology, Pain and Perioperative Medicine, The First Affiliated Hospital of Zhengzhou University, Zhengzhou, Henan China; 2 Department of Anesthesiology, Zhongda Hospital, School of Medicine, Southeast University, Nanjing, Jiangsu China

**Keywords:** (*2R,6R*)-hydroxynorketamine, Antidepressant, Brain-derived neurotrophic factor, Narp, Hippocampus

## Abstract

**Background:**

Preclinical studies have indicated that the ketamine metabolite (*2R,6R*)-hydroxynorketamine (HNK) is a rapid-acting antidepressant drug with limited dissociation properties and low abuse potential. However, its effects and molecular mechanisms remain unclear. In this work, we examined the involvement of brain-derived neurotrophic factor (BDNF), tropomyosin receptor kinase B (TrkB) and Narp in the antidepressant-like actions of (*2R,6R*)-HNK in a chronic restraint stress (CRS) mouse model.

**Methods:**

C57BL/6 male mice were subjected to CRS for 8 h per day for 14 consecutive days. Open field, forced swimming, novelty suppressed feeding, and tail suspension tests were performed after administering (*2R,6R*)-HNK (10 mg/kg), a combination of (*2R,6R*)-HNK and NBQX (an alpha-amino-3-hydroxy-5-methyl-4-isoxazole propionic acid (AMPA) receptor antagonist; 10 mg/kg), or a combination of (*2R,6R*)-HNK and ANA-12 (a TrkB receptor antagonist; 0.5 mg/kg). The mRNA levels of *Bdnf* and *Narp* in the hippocampus were determined by quantitative reverse transcription-PCR (qRT–PCR). Western blotting was used to determine the hippocampal protein levels of GluA1, GluA2, BDNF, Narp, PSD95, and synaptophysin, as well as the p-TrkB/TrkB protein ratio.

**Results:**

(*2R,6R*)-HNK had rapid antidepressant-like effects in CRS mice. Furthermore, (*2R,6R*)-HNK significantly ameliorated CRS-induced downregulation of GluA1, GluA2, BDNF, Narp, PSD95, and the p-TrkB/TrkB protein ratio in the hippocampus. The effects of (*2R,6R*)-HNK were blocked by combinations with NBQX or ANA-12.

**Conclusion:**

BDNF-TrkB signaling-mediated upregulation of Narp in the hippocampus may play a key role in the antidepressant-like effect of (*2R,6R*)-HNK in the CRS model of depression.

**Supplementary Information:**

The online version contains supplementary material available at 10.1186/s12888-022-03838-x.

## Introduction

Major depressive disorder (MDD) is a chronic and debilitating mental disorder that affects over 264 million people worldwide and causes serious health and socioeconomic consequences [1]. Current monoaminergic-based pharmacotherapies often take several weeks or months to alleviate clinical symptoms [2]. In addition, treatment resistance and nonresponse rates of up to 30% have made these current treatments less reliable [2]. Laboratory and clinical studies have provided strong evidence for the rapid-acting (within hours) and sustained (lasting up to 7 days) antidepressant-like actions of (*R,S*)-ketamine (ketamine), an N-methyl-D-aspartate (NMDA) receptor antagonist, in treatment-resistant patients with MDD [3–6]. Although ketamine is a promising alternative to standard clinically prescribed drugs and is regarded as one of the most significant advances in psychiatry in recent decades, its dissociative properties, changes in sensory perception, and abuse liability [7] have prompted a search for alternative compounds that trigger robust antidepressant-like effects without inducing psychotomimetic side effects.

Recently, one of the ketamine metabolites, (2*R*,6*R*)-hydroxynorketamine (HNK), has been proposed as an ideal next-generation agent, as it has strikingly rapid and robust antidepressant-like effects without the adverse effects of ketamine [8–13]. This interesting metabolite has been reported to function as an antidepressant in animal models by enhancing the expression and function of α-amino-3-hydroxy-5-methyl-4-isoxazolepropionic acid (AMPA) receptors in the hippocampus [8, 10, 13], and it has piqued the interest of researchers to determine its clinical efficacy in depressed patients and to investigate its underlying mechanisms.

The activity-dependent release of brain-derived neurotrophic factor (BDNF) and the activation of downstream tropomyosin receptor kinase B (TrkB) receptors in the hippocampus play critical roles in the antidepressant-like effects of ketamine and its metabolites [8, 14, 15]. The stimulation of BDNF-TrkB signaling promotes the transcription of many synaptic genes and increases the number and function of synapses [16]. Neuronal activity-regulated pentraxin 2 (Narp) is highly expressed in the hippocampus and cortex and is associated with excitatory synaptogenesis and AMPA receptor aggregation [17, 18]. There is some evidence that BDNF expression and Narp expression are related [5, 19, 20]. Mariga *et al*. demonstrated that BDNF directly regulates Narp, mediating glutamatergic transmission and mossy fiber plasticity in the hippocampus [5].

In this work, we sought to investigate whether the ketamine metabolite (*2R,6R*)-HNK rescues chronic restraint stress (CRS)-induced depression-like behavior through upregulation of AMPA receptors expression mechanisms. We also investigated the role of BDNF-mediated Narp expression in the antidepressant-like effects of (*2R,6R*)-HNK.

## Methods and materials

### Animal groups

**Fig. 1:**
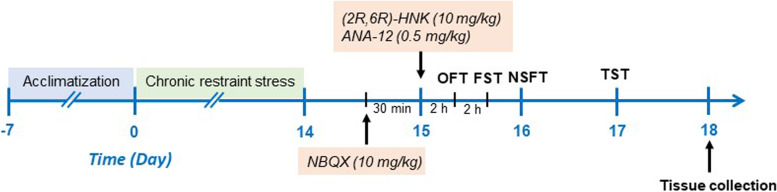
Timeline of drug injection, behavioral tests, and tissue collection. See the text for details. OFT, open field test; FST, forced swimming test; NSFT, novelty suppressed feeding test; TST, tail suspension test

All experimental procedures were approved by the Ethics Committee of Zhongda Hospital, Medical School, Southeast University. All animal experiments were carried out in strict accordance with the National Institutes of Health Guide for the Care and Use of Laboratory Animals. Eighty male 7-week-old C57BL/6J mice were purchased from the Animal Center of Southeast University, Nanjing, China. This initial study was carried out in male mice to control for hormonal variables, as ovarian hormones are necessary for the efficacy of (*2R,6R*)-HNK in females [21]. Animals were housed in groups of four per cage under controlled illumination (12 h light/dark cycle, lights on 07:00 to 19:00) and temperature (23 ± 1 °C) with free access to food and water. After one week of acclimation, the mice were randomly divided into five groups (n = 16): the control group, the CRS group, the CRS plus HNK (Sigma–Aldrich, St. Louis, MO, USA) group, the CRS plus HNK plus NBQX (an AMPA receptor antagonist; Tocris Bioscience, Bristol, UK) group, and the CRS plus HNK plus ANA-12 (a noncompetitive TrkB receptor antagonist; Maybridge Chemical Company, Tintagel, UK) group. Mice were decapitated after the behavioral tests, and the hippocampus was rapidly dissected, frozen, and stored at -80 °C for further use. The schematic timeline of the experimental procedure is summarized in Fig. 1.

### Chronic restraint stress

For restraint stress, 8-week-old mice weighing 22–23 g were individually placed head-first into a well-ventilated 50 ml polypropylene conical tube, and their tails were removed through a 3 cm long tube and a small hole in the cap of the tube. Mice could not move forward or backward in this device. This restraint stress was administered to animals daily for 8 h, from 9 am to 5 pm, for 14 consecutive days [22, 23]. The control mice remained undisturbed in their original cages in their home environment. The stressed animals were returned to their home environment following the session of restraint stress.

### Drugs

All drugs were administered intraperitoneally. For (*2R,6R*)-HNK administration, fresh solutions (10 mg/kg) were prepared with 0.9% saline and administered on Day 15. NBQX (10 mg/kg) was dissolved in 0.9% saline and administered to mice intraperitoneally 30 min before *(2R,6R)*-HNK. ANA-12 (0.5 mg/kg) was prepared in vehicle containing 1% dimethyl sulfoxide and coadministered with (*2R,6R*)-HNK to mice. The selected doses and injection time points were based on previous studies [13, 24].

### Open field test (OFT)

Exploration in response to a novel open field was measured 2 h after (*2R,6R*)-HNK administration. Animals were placed in the center of an arena (50 cm × 50 cm × 40 cm; length × width × height) in a dimly lit room and allowed to move freely for 5 min. A video camera positioned directly above the arena was used to track the movement of each animal with software (XR-XZ301, Shanghai Softmaze Information Technology Co., Ltd., Shanghai, China). The dependent measurements were the total distance traveled, the time spent in the center, and the number of entries into the center.

### Forced swimming test (FST)

The test was performed 4 h after (*2R,6R*)-HNK administration to evaluate depression-like behavior. Mice were placed in a glass container (20 cm diameter × 30 cm height) filled to a depth of 15 cm with water (23-25 °C) and allowed to swim for 6 min. The immobility time during the last 4 min was measured by an observer blinded to animal treatment. The immobility time refers to the time when a mouse floated passively with no additional activity or movements other than those required to maintain balance in the water [25]. After the experiment, the mouse body was wiped dry with absorbent paper, and the mouse was returned to its the original cage. The water was replaced at the end of each test.

### Novelty suppressed feeding test

The novelty suppressed feeding test (NSFT) was carried out according to previous studies [26, 27]. The testing apparatus was a plastic box (50 cm × 50 cm × 40 cm; length × width × height), the floor of which was covered with approximately 2 cm of wooden bedding. The mice were housed alone in freshly made home cages and food deprived 24 h prior to behavioral testing. At the time of testing, a single food pellet was placed on a white paper platform in the center of the box. An animal was placed in a corner of the box, and the time needed for the mice to consume some food (the feeding latency) was recorded by a trained observer. Immediately afterward, the animal was returned to its home cage, which contained preweighed food pellets, and the amount of food consumed by the mouse during the next 5 min was measured. Each mouse was weighed before food deprivation and before testing to assess the percentage of body weight loss.

### Tail suspension test (TST)

Mice were suspended by their tails and secured to a horizontal bar with tape. The immobility time was recorded for 6 min. Mice were considered immobile only when they hung passively and were completely motionless [26]. The behavioral apparatus was thoroughly cleaned with 70% ethanol between animals.

### Quantitative mRNA measurements

We analyzed the mRNA levels of *Bdnf* and *Narp* in the hippocampus via quantitative reverse transcription-PCR (qRT–PCR) in a StepOnePlus™ Real-Time PCR System (Applied Biosystems, Foster City, CA), as previously described [28]. We extracted RNA from the samples using an RNeasy Plus Kit (Qiagen, Valencia, CA), reverse-transcribed it with a high-capacity cDNA reverse transcription kit (Bio–Rad Laboratories, Hercules, CA), and then analyzed it with qRT–PCR. TaqMan probes for *Bdnf* (Mm04230607_s1) and *Narp* (Mm00479438_m1) were obtained from Applied Biosystems (Carlsbad, CA, USA). Data were normalized to glyceraldehyde-3-phosphate dehydrogenase (*Gapdh*) mRNA (Mm99999915_g1). The gene expression was calculated using the ΔΔCT method [29], and data are presented as the relative fold change from control animals.

### Western blotting analysis

The hippocampus was homogenized in an RIPA lysis buffer mixed with 1% protease inhibitor cocktail and 1% phenylmethanesulfonyl fluoride. After centrifugation at 13,000 g for 10 min at 4 °C, the supernatant was collected, and the protein concentration was determined by a BCA protein assay kit (Beyotime, China). Forty micrograms of protein per lane was loaded on SDS–PAGE gels and then transferred to polyvinylidene fluoride membranes. After blocking with 3% bovine serum albumin in Tris Buffered Saline with Tween (TBST) for 1 h at room temperature, the membranes were incubated at 4 °C overnight with primary antibodies, including recombinant anti-GluA1 (1:1000; ab109450, Abcam, Cambridge, UK), recombinant anti-GluA2 (1:1000; ab133477, Abcam, Cambridge, UK), rabbit anti-BDNF (1:1000; ab226843, Abcam, Cambridge, UK), recombinant anti-TrkB (phospho Y705) (1:1000; ab229908, Abcam, Cambridge, UK), rabbit anti-TrkB (1:1000; 4606S, Cell Signaling, Danvers, MA, USA), recombinant anti-Narp (1:1000; ab277523, Abcam, Cambridge, UK), rabbit anti-postsynaptic density 95 (PSD95) (1:1000; ab18258, Abcam, Cambridge, UK), recombinant anti-synaptophysin (1:1000; ab32127, Abcam, Cambridge, UK), and mouse anti-β-actin (1:1000; ab8226, Abcam, Cambridge, UK). The membranes were washed three times in TBST before being incubated for 1 h at room temperature with goat anti-rabbit or mouse IgG-horseradish peroxidase-conjugated secondary antibodies (1:7000, Bioworld Technology, St. Louis Park, MN, USA). The protein bands were detected by enhanced chemiluminescence, exposed to X-ray film, and quantitated with ImageJ software (National Institutes of Health, Bethesda, MD, USA).

### Statistical analysis

Statistical analyses were conducted on raw data using SigmaPlot 14.0 software (Systat Software Inc., San Jose, CA, USA), which automatically determined whether the dataset met test criteria (Shapiro–Wilk for the normality test and Brown-Forsythe for the equal variance test), and Prism V8.0 software (GraphPad, San Diego, CA, USA). Data are presented as the mean ± SEM. The differences between the groups were compared using one-way analysis of variance (ANOVA) followed by *post hoc* Holm-Sidak tests. *P* < 0.05 was considered significant.

## Results

### Effects of (2R,6R)-HNK on CRS-induced depression-like behaviors

**Fig. 2:**
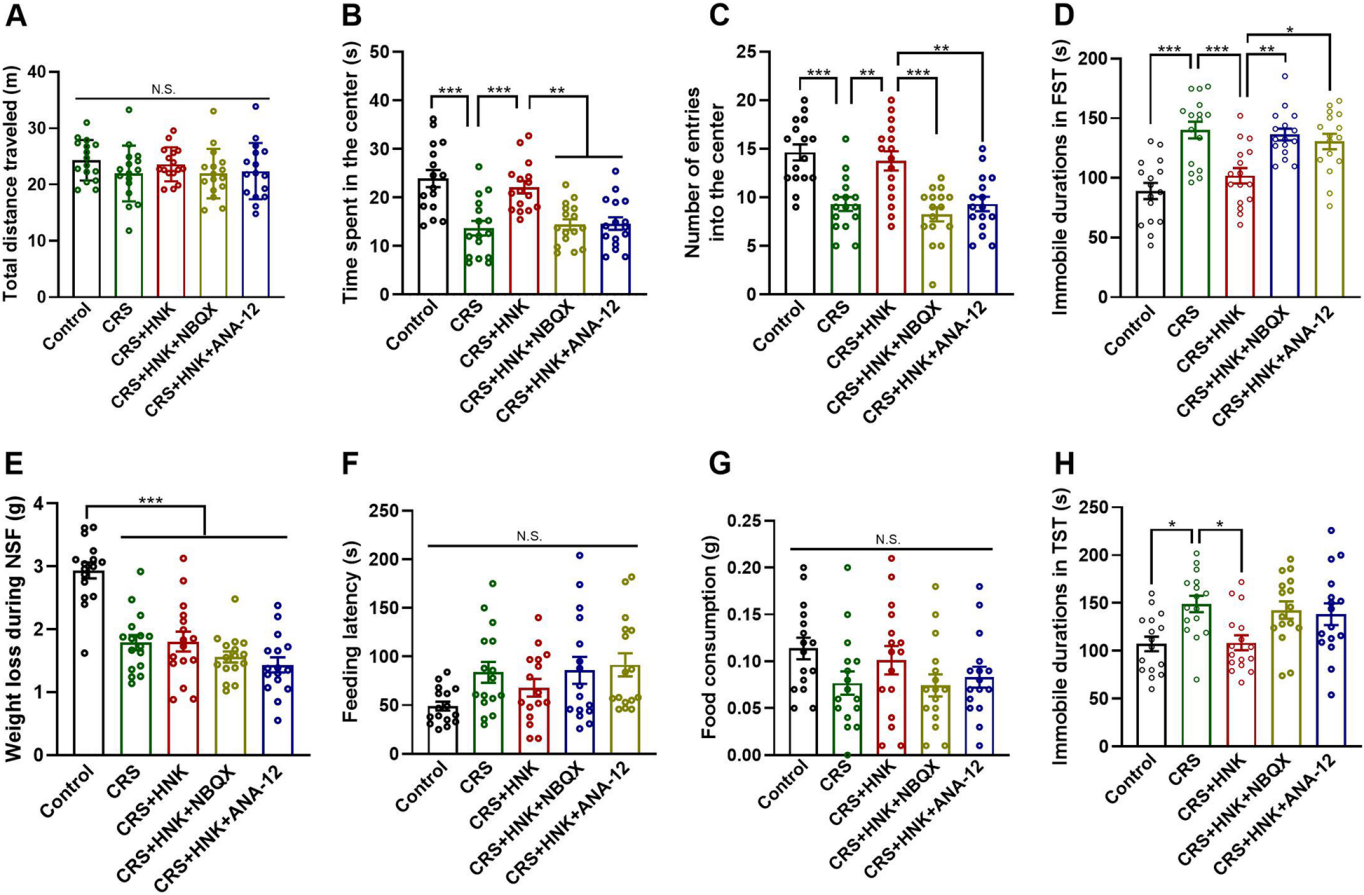
Effects of (*2R,6R*)-HNK on chronic restraint stress (CRS)-induced depression-like behaviors. **A**-**C** Histograms showing the total distance traveled, time spent in the center, and number of entries into the center of the open field by mice during the open field test. **D** Histogram showing the immobility time of the mice in the forced swimming test (FST). **E**-**G** Histograms showing weight loss during the fast for the novelty suppressed feeding test (NSFT), the feeding latency, and the total food consumption in the NSFT. **H** Histogram showing the immobility time of the mice in the tail suspension test (TST). Data are presented as the mean ± SEM (n = 16 mice per group). *P < 0.05, **P < 0.01, and ***P < 0.001. N.S., not significant

In the OFT, there was a significant difference between subjects from different groups in terms of the time they spent in the center (F_(4,75)_ = 12.016, *P* < 0.001, Fig. 2B) and the number of entries into the center (F_(4,75)_ = 13.148, *P* < 0.001, Fig. 2C), but not in terms of the distance traveled during the test (F_(4,75)_ = 0.993, *P* = 0.417; Fig. 2A). Specifically, mice in the CRS group spent less time in the center and made fewer entries into the center than mice in the control group (time spent in the center: *P* < 0.001; entries into the center: *P* < 0.001) and mice in the CRS + HNK group (time spent in the center: *P* < 0.001; entries into the center: *P* = 0.001). Compared with mice administered (*2R,6R*)-HNK only, the time spent in the center and the number of entries into the center were decreased in mice pretreated with NBQX (time spent in the center: *P* = 0.001; entries into the center: *P* < 0.001) or cotreated with ANA-12 (time spent in the center: *P* = 0.002; entries into the center: *P* = 0.001).

In the FST, there was a significant difference in immobility time between treatment groups (F_(4,75)_ = 12.810, *P* < 0.001, Fig. 2D). Multiple pairwise comparisons revealed that mice in the CRS group had longer immobility time than mice in the control group (*P* < 0.001) and mice in the CRS + HNK group (*P* < 0.001). The immobility times in the CRS + HNK + NBQX group (*P* = 0.002) and the CRS + HNK+ ANA-12 group (*P* = 0.011) were longer than that in the CRS + HNK group.

Mice in the control group lost more weight than mice in all other groups during the fast for the NSFT (F_(4,75)_ = 23.082, *P* < 0.001, Fig. 2E). One-way ANOVA revealed that treatment had a significant effect on feeding latency (F_(4,75)_ = 2.704, *P* = 0.037, Fig. 2F). However, the pairwise multiple comparison analysis did not show a significant difference between groups. The total food consumption was unaffected across all five groups (F_(4,75)_ = 1.849, *P* = 0.128, Fig. 2G).

In the TST, there was a significant difference in immobility time between treatment groups (F_(4,75)_ = 4.878, *P* = 0.001, Fig. 2H). Multiple pairwise comparisons revealed that the mice in the CRS group had significantly longer immobility time than the mice in the control (*P* = 0.015) and CRS + HNK (*P* = 0.020) groups. However, pretreatment with NBQX (*P* = 0.944 when compared to the CRS group) and coadministration with ANA-12 (*P* = 0.872 when compared to the CRS group) blocked the antidepressant-like action of (*2R,6R*)-HNK in the TST.

### Roles of the mRNA levels of hippocampal Bdnf and Narp in the antidepressant-like activity of (2R,6R)-HNK

**Fig. 3:**
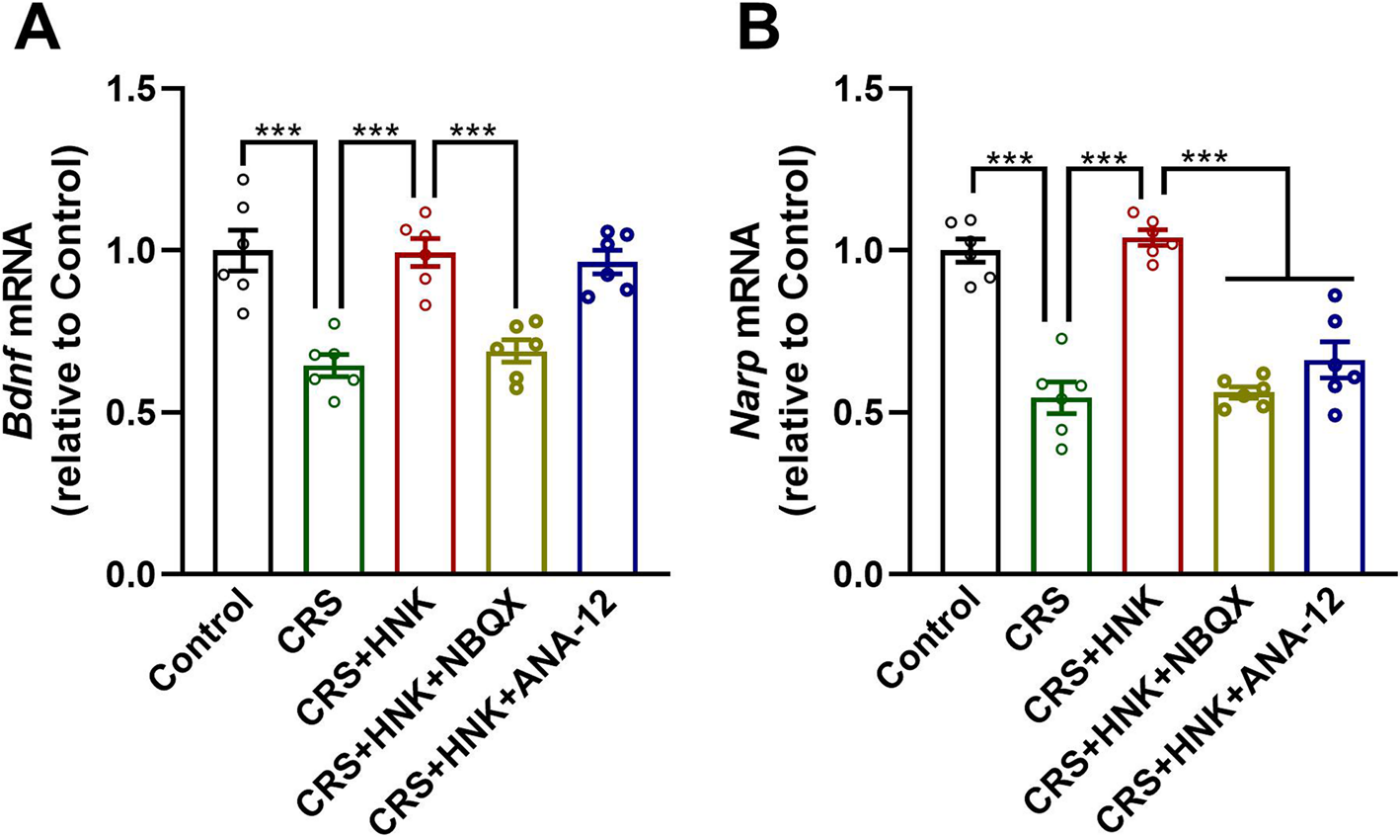
Role of the mRNA levels of hippocampal *Bdnf* and *Narp* in the antidepressant-like activity of (2R,6R)-HNK. **A**, **B** The respective mRNA levels of *Bdnf* and *Narp* in the hippocampi of mice. Data are shown as the mean ± SEM, with 6 mice/group. ***P < 0.001

In the hippocampal gene transcription measurements, there was a statistically significant difference in the hippocampal mRNA levels of *Bdnf* between treatment groups (F_(4,25)_ = 16.184, *P* < 0.001, Fig. 3A). The pairwise multiple comparison analysis revealed that, when compared with the CRS group, (*2R,6R*)-HNK ameliorated the CRS-induced decrease in *Bdnf* mRNA levels (*P < 0.001*), which was abolished by injection with NBQX (*P* < 0.001) but not ANA-12 (*P* = 0.876). Similarly, there was a statistically significant difference in the hippocampal Narp mRNA levels of subjects that received different treatments (F_(4,25)_ = 37.831, *P* < 0.001, Fig. 3B). The pairwise multiple comparison analysis showed that, when compared with the CRS group, (*2R,6R*)-HNK ameliorated the CRS-induced decrease in *Narp* transcription (*P < 0.001*), which was abolished by both pretreatment with NBQX (*P < 0.001*) and cotreatment with ANA-12 (*P < 0.001*).

### Roles of the protein levels of hippocampal GluA1, GluA2, BDNF, Narp, PSD95 and synaptophysin and the p-TrkB/TrkB protein ratio in the antidepressant-like activity of (2R,6R)-HNK

**Fig. 4:**
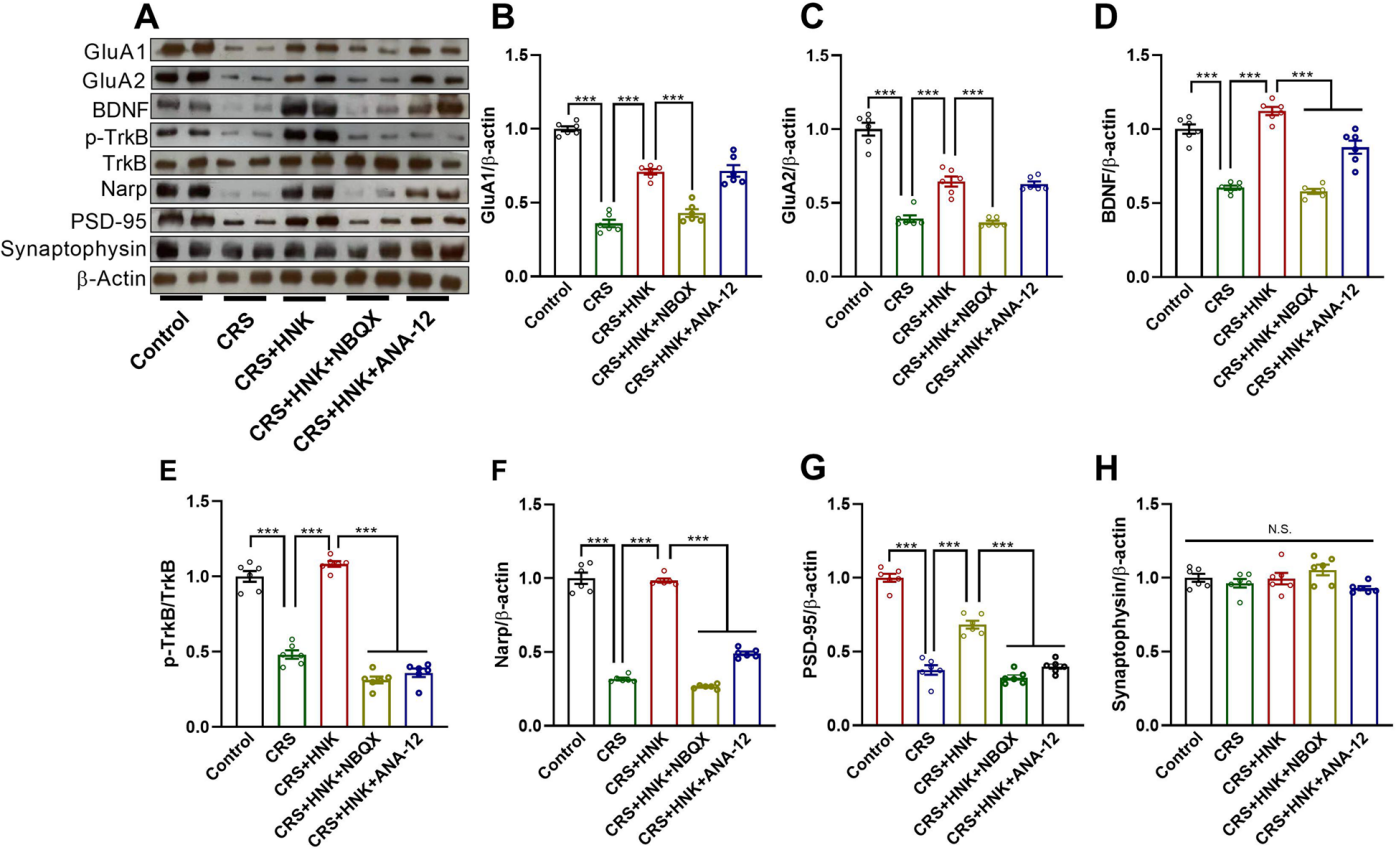
Roles of AMPA receptors and BDNF-TrkB signaling in the antidepressant-like activity of (2R,6R)-HNK. **A** Representative Western blots of the protein levels in the five groups. Full-length blots are presented in Supplementary Figure 1, 2, 3. **B**–**H** The respective protein levels of GluA1, GluA2, BDNF, the p-TrkB/TrkB protein ratio, Narp, PSD95, and synaptophysin in the hippocampi of male mice. Data, normalized against the control, are shown as the mean ± SEM, with 6 mice/group. ***P < 0.001. N.S., not significant

To investigate the role of AMPA receptors in the antidepressant-like actions of (*2R,6R*)-HNK, we determined the protein levels of hippocampal GluA1 and GluA2, which are important subunits of AMPA receptors. We found statistically significant differences in the hippocampal GluA1 and GluA2 protein levels between treatment groups (GluA1: F_(4,25)_ = 93.635, *P* < 0.001, Fig. 4A, B; GluA2: F_(4,25)_ = 80.131, *P* < 0.001, Fig. 4A, C). The pairwise multiple comparison analysis showed that GluA1 and GluA2 protein levels in the hippocampi of CRS mice were significantly lower than those in control mice (*P* < 0.001). (*2R,6R*)-HNK significantly increased GluA1 and GluA2 levels in the hippocampus (*P* < 0.001), and this increase was abolished by pretreatment with NBQX (*P* < 0.001) but not by cotreatment with ANA-12 (GluA1: *P* = 0.879; GluA2: *P* = 0.670). As BDNF-TrkB signaling is a putative pathway in the therapeutic action of (*2R,6R*)-HNK, we performed Western blotting analyses of BDNF protein levels and the p-TrkB/TrkB ratio. There were statistically significant differences in BDNF protein levels (F_(4,25)_ = 68.315, *P* < 0.001, Fig. 4A, D) and the p-TrkB/TrkB protein ratio (F_(4,25)_ = 176.748, *P* < 0.001, Fig. 4A, E) between treatment groups. The pairwise multiple comparison analysis showed that the BDNF protein levels in the hippocampi of CRS mice were significantly lower than those in control mice (*P* < 0.001). (*2R,6R*)-HNK significantly increased BDNF levels (*P* < 0.001), and this increase was abolished by pretreatment with NBQX (*P* < 0.001) or cotreatment with ANA-12 (*P* < 0.001). Compared with the p-TrkB/TrkB ratio in the control and CRS + HNK groups, the p-TrkB/TrkB ratio in the CRS group was decreased (*P* < 0.001). Pretreatment with NBQX or cotreatment with ANA-12 abolished the (*2R,6R*)-HNK-induced increase in the p-TrkB/TrkB ratio in CRS mice (*P* < 0.001). One-way ANOVA of Narp protein levels revealed a significant difference among the five groups (F_(4,25)_ = 176.748, *P* < 0.001, Fig. 4A, F). The pairwise multiple comparison analysis showed that Narp protein levels in the hippocampi of CRS mice were significantly lower than those in control mice (*P* < 0.001). (*2R,6R*)-HNK significantly increased Narp levels (*P <* 0.001), and this increase was abolished by pretreatment with NBQX or cotreatment with ANA-12 (*P <* 0.001). The treatment also had a statistically significant effect on PSD95 protein levels (F_(4,25)_ = 129.127, *P <* 0.001; Fig. 4A, H), but not on the levels of the presynaptic membrane protein synaptophysin (F_(4,25)_ = 2.301, *P* = 0.087; Fig. 4A, G). Consistent with these results, the pairwise multiple comparison analysis revealed that the PSD95 protein levels in the CRS group were lower than those in the control and CRS + HNK groups (*P <* 0.001). The PSD95 protein levels were lower in mice pretreated with NBQX (*P <* 0.001) or cotreated with ANA-12 (*P <* 0.001) than in mice administered (*2R,6R*)-HNK only.

## Discussion

The novel finding of this study is that increased AMPA receptors and BDNF expression, activation of downstream TrkB receptors, which resulted in increased Narp expression, are associated with the antidepressant-like effects of (*2R,6R*)*-*HNK. The antidepressant-like effects of (*2R,6R*)*-*HNK are blocked by the AMPA receptor antagonist NBQX and the TrkB antagonist ANA-12. Overall, these results suggest that the BDNF-TrkB signaling-mediated upregulation of Narp plays a key role in the antidepressant-like effects of (*2R,6R*)*-*HNK by influencing synaptic plasticity.

Chronic stress is a risk factor for psychiatric illnesses such as anxiety and depression [30, 31]. For this reason, we established a chronic restraint stress animal model of depression, which has been well described in previous studies [22, 23]. Kim et al. demonstrated that restraint treatment for 8 h per day for 14 days successfully produced anxiety- and depression-like behaviors, whereas restraint treatment for 2 h per day for 10 days was only marginally effective [22]. In our study, CRS-induced depression-like behaviors were reversed by (*2R,6R*)*-*HNK administration, confirming previous observations of the antidepressant-like effects of (*2R,6R*)*-*HNK [8, 10, 12, 13, 32]. However, (*2R,6R*)-HNK has also been reported to lack antidepressant effects or exert have poor antidepressant effects in chronic social-defeat stress (CSDS), lipopolysaccharide (LPS), chronic corticosterone, and learned helplessness (LH) models [33–35]. The reason for the discrepancy between these findings remains unclear, but it could partially be due to differences in the strain, species, animal models of depression, behavioral test procedures, or drug doses. A clinical trial of (*2R,6R*)-HNK for therapeutic efficacy in humans is ongoing at the United States National Institute for Mental Health [36]. It is of great interest to explore the antidepressant-like effects of (*2R,6R*)-HNK in MDD patients.

Currently, the precise mechanisms underlying the effects of (*2R,6R*)-HNK are still unknown. AMPA receptors play a role in the antidepressant-like activity of ketamine [37]. Ketamine-induced glutamate release activates AMPA receptors by acting on NMDA receptors, resulting in the synthesis and release of BDNF [38], which has been identified as an important mediator of synaptic plasticity [39]. Multiple studies have suggested that BDNF-TrkB signaling is important in the pathophysiology of depression and as a therapeutic target for antidepressants [8, 14, 15]. In this study, we found a marked increase in the AMPA receptor subunits GluA1 and GluA2 in the hippocampus after (*2R,6R*)-HNK administration in CRS mice, whereas pretreatment with NBQX significantly blocked the effects of (*2R,6R*)-HNK, which is consistent with the recent finding that (2*R*,6*R*)-HNK administration induces the upregulation of synaptic AMPA receptors [13, 29]. The results provide evidence that (*2R,6R*)-HNK rescues chronic stress-induced depression-like behavior through increased AMPA receptors expression in the hippocampus. Fukumoto et al. found that the antidepressant-like actions of (*2R,6R*)-HNK were inhibited by knocking in the BDNF Val66Met allele (which blocks the processing and release of BDNF) or by injecting an anti-BDNF antibody into the medial prefrontal cortex (mPFC), demonstrating (*2R,6R*)-HNK induces long-lasting antidepressant behavioral responses via activity-dependent BDNF release [8]. A recent study also reported that the administration of a neutralizing BDNF antibody or inhibitors of the BDNF signaling pathway in the ventrolateral periaqueductal gray 30 min before the (*2R,6R*)-HNK treatment blocked the actions of (*2R,6R*)-HNK. However, the BDNF RNAi attenuated the actions of (2R,6R)-HNK [40]. As shown in the present study, the BDNF levels and the p-TrkB/TrkB ratio in the hippocampi of CRS mice both increased after (*2R,6R*)-HNK administration. The antidepressant-like effects of (*2R,6R*)-HNK were blocked by ANA-12, a TrkB receptor antagonist. Taken together, these findings suggest that AMPA receptor-driven BDNF-TrkB signaling plays a contributing role in mediating the antidepressant-like effects of (*2R,6R*)-HNK.

It has been reported that Narp is a direct transcriptional target of BDNF [5]. Acute BDNF withdrawal results in the downregulation of Narp, whereas BDNF greatly increases Narp transcription [5]. Furthermore, Narp knockout mice exhibit anxiety- and depression-like behaviors [16, 41]. The selective serotonin reuptake inhibitor (SSRI) fluoxetine increased hippocampal Narp mRNA expression in healthy control rats [42]. These findings suggest that Narp plays a role in BDNF-induced antidepressant activity. In this study, we found that Narp expression in the hippocampi of CRS mice increased after a single injection of (*2R,6R*)-HNK, and its expression could be blocked by coadministration with ANA-12. Thus, CRS may cause a decrease in BDNF levels in the hippocampus, which leads to a decrease in Narp expression, resulting in depression-like behaviors in mice, whereas (*2R,6R*)-HNK exerts its antidepressant-like actions via BDNF/TrkB receptors and the Narp pathway in the hippocampus.

The mechanism by which Narp mediates the therapeutic activity of antidepressants is still unknown. Narp, which is expressed by pyramidal neurons and secreted by axon terminals, uniquely mediates the activity-dependent strengthening of pyramidal neuron excitatory synapses by promoting AMPA receptor accumulation [42]. Overexpression of Narp results in synaptic targeting and AMPA receptor stabilization at excitatory synapses [43]. Furthermore, Narp can promote neuronal migration and dendritic neurite outgrowth, and it is upregulated following long-term potentiation (LTP) induction [44]. These features underlie the contributions of Narp to synaptic plasticity, which is considered to be one of the neurobiological mechanisms of depression [45]. We found that (*2R,6R*)-HNK administration in CRS mice increased PSD95 protein levels, which is involved in synaptic plasticity and the anchoring of synaptic proteins [46]. Studies on the enhanced synaptic function of spine synapses have provided additional evidence that synaptic function is involved in the actions of (*2R,6R*)-HNK [8, 13]. More mechanistic research is needed in the future, such as observing spine morphology changes and applying brain-specific gene deletion or overexpression methods to investigate the role of Narp in the antidepressant-like effects of (*2R,6R*)-HNK.

In summary, these results demonstrate that (*2R,6R*)-HNK induces robust antidepressant behavioral responses by stimulating the BDNF-TrkB signaling pathway, which further increases Narp expression. The current results provide critical insights into the mechanism of action of (*2R,6R*)-HNK, which will aid in the development of more effective and safer antidepressants.

## Supplementary Information

**Additional file 1.**

**Additional file 2.**

**Additional file 3.**

## Data Availability

The datasets analyzed during the current study are not publicly available but are available from the corresponding author on reasonable request.
