# Supplementary figures and images for: BDNF-TrkB signaling-mediated upregulation of Narp is involved in the antidepressant-like effects of (2R,6R)-hydroxynorketamine in a chronic restraint stress mouse model
Source: BMC Psychiatry. 2022 Mar 15;22:182. doi: 10.1186/s12888-022-03838-x (PMC8922900; doi:10.1186/s12888-022-03838-x)

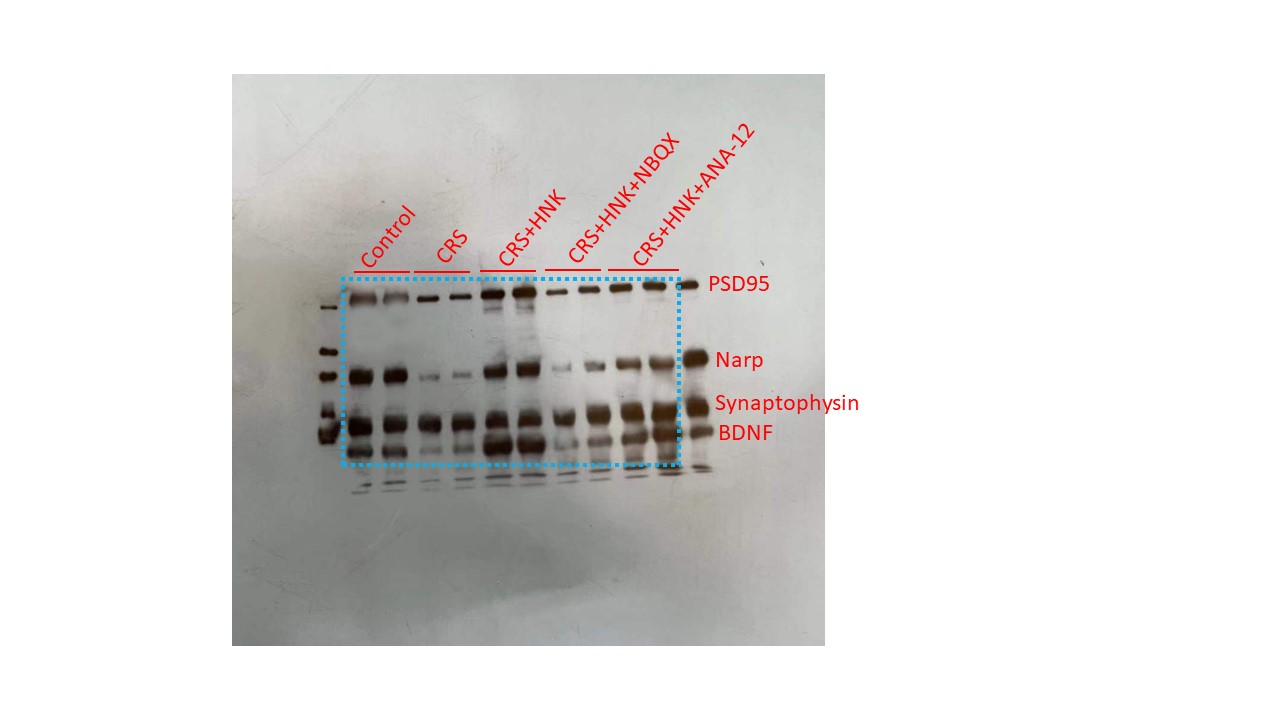

Supplement: Supplementary file 1 — Additional file 1. [file 12888_2022_3838_MOESM1_ESM.jpg]

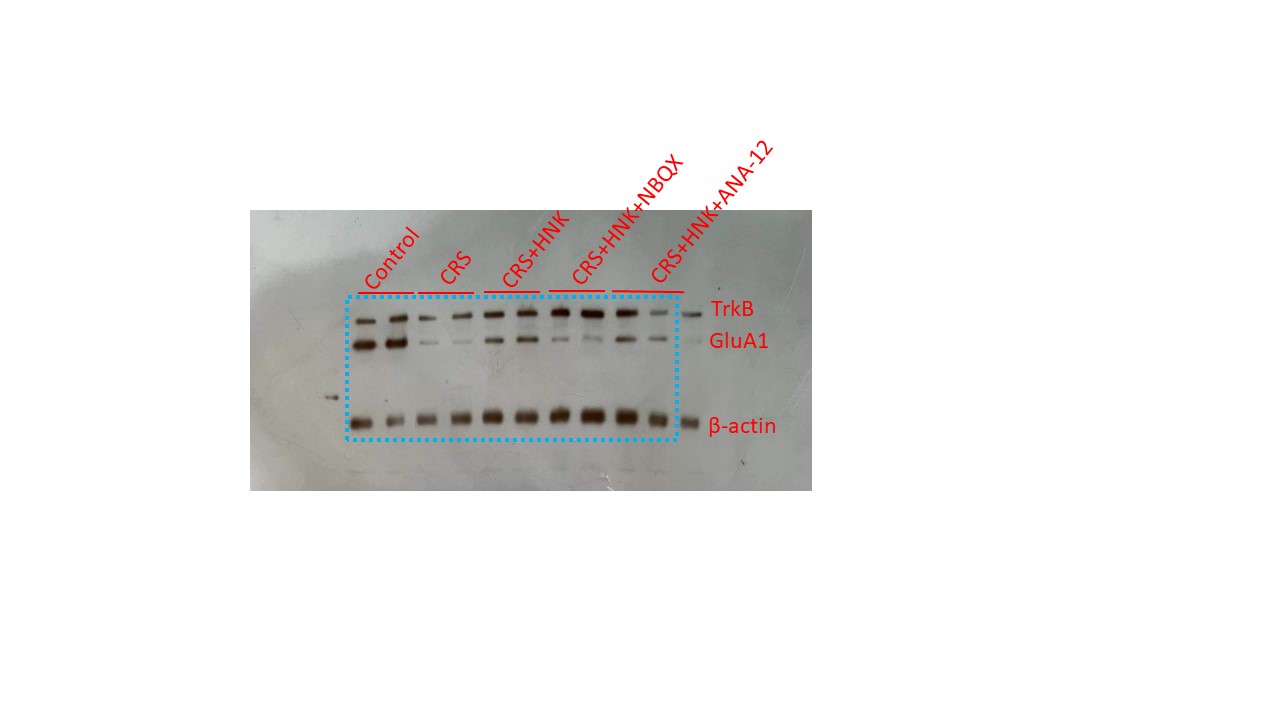

Supplement: Supplementary file 2 — Additional file 2. [file 12888_2022_3838_MOESM2_ESM.jpg]

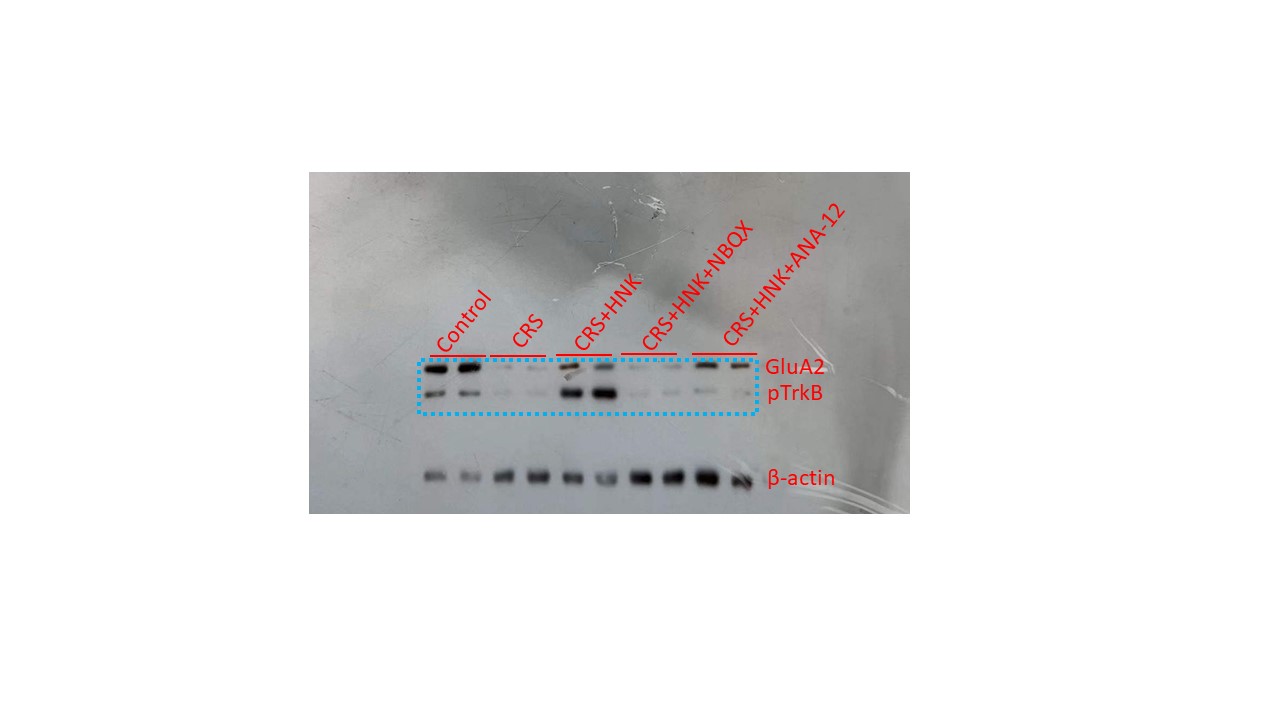

Supplement: Supplementary file 3 — Additional file 3. [file 12888_2022_3838_MOESM3_ESM.jpg]
